# The Role of Environmental And Spatial Factors For Person-Centred Care In Residential Long-Term Care Facilities

**DOI:** 10.1093/geroni/igaf122.3039

**Published:** 2025-12-31

**Authors:** Oscar Ribeiro, Miguel Padeiro, Flavia Borges-Machado, Liliana Sousa, Paula Santana

**Affiliations:** University of Aveiro, Aveiro, Aveiro, Portugal; University of Coimbra, Coimbra, Coimbra, Portugal; University of Aveiro, Aveiro, Aveiro, Portugal; University of Aveiro, Aveiro, Aveiro, Portugal; University of Coimbra, Coimbra, Coimbra, Portugal

## Abstract

This cross-sectional study investigated variations in person-centred care (PCC) across Portuguese residential long-term care facilities, focusing on environmental and spatial determinants, including facility location, surrounding local environment, and outdoor spaces. Data were collected through a self-administered anonymous online survey distributed nationwide to care home directors between January and June 2024. PCC was assessed using the Person-Centred Care Assessment Tool (P-CAT), and statistical analyses, including bivariate and multivariate models, were conducted using RStudio. A total of 424 directors participated, with the majority representing non-profit cooperative and social sector entities (81.56%). Facilities had an average of 39.63 residents, of whom approximately one-third (31.66%) were people living with dementia (PLwD). P-CAT scores ranged from 34 to 65. Higher PCC levels were positively associated with a perceived sense of security in the local environment, the presence of local commerce, and perceived neighborhood quietness. Concerning outdoor spaces, a sense of security and the presence of an orchard or vegetable garden were also significant predictors of PCC. These findings highlight the relevance of environmental and spatial factors, which are often overlooked in existing research, emphasizing the need to integrate territorial and external contextual variables when assessing PCC variations in long-term care settings. Funding by Fundação para a Ciência e a Tecnologia: SINDIA (2022.04684.PTDC), CEGOT (UIDB/04084/2020), RISE (LA/P/0053/2020).

